# Stacked thin layers of metaphase chromatin explain the geometry of chromosome rearrangements and banding

**DOI:** 10.1038/srep14891

**Published:** 2015-10-08

**Authors:** Joan-Ramon Daban

**Affiliations:** 1 Departament de Bioquímica i Biologia Molecular, Facultat de Biociències, Universitat Autònoma de Barcelona, 08193-Bellaterra, Spain

## Abstract

The three-dimensional organization of tightly condensed chromatin within metaphase chromosomes has been one of the most challenging problems in structural biology since the discovery of the nucleosome. This study shows that chromosome images obtained from typical banded karyotypes and from different multicolour cytogenetic analyses can be used to gain information about the internal structure of chromosomes. Chromatin bands and the connection surfaces in sister chromatid exchanges and in cancer translocations are planar and orthogonal to the chromosome axis. Chromosome stretching produces band splitting and even the thinnest bands are orthogonal and well defined, indicating that short stretches of DNA can occupy completely the chromosome cross-section. These observations impose strong physical constraints on models that attempt to explain chromatin folding in chromosomes. The thin-plate model, which consists of many stacked layers of planar chromatin perpendicular to the chromosome axis, is compatible with the observed orientation of bands, with the existence of thin bands, and with band splitting; it is also compatible with the orthogonal orientation and planar geometry of the connection surfaces in chromosome rearrangements. The results obtained provide a consistent interpretation of the chromosome structural properties that are used in clinical cytogenetics for the diagnosis of hereditary diseases and cancers.

In the cell nucleus, genomic DNA molecules are associated with histone proteins and form long chromatin filaments containing many nucleosomes. The core particle of each nucleosome is a short cylinder (5.7 nm height and 11 nm diameter), which consist of a segment of 146 bp of DNA wrapped around a histone octamer^1^. Chromatin filaments are dynamic structures that can have different compaction degrees, ranging from extended fibres up to compact interdigitated solenoids (~30 nm diameter)^2^,^3^,^4^,^5^,^6^. During mitosis, each sister chromatid of a condensed chromosome contains a single DNA molecule that is densely packaged^7^. Metaphase chromatids of different plant and animal species show a great variety of sizes which are dependent on the amount of DNA that they contain, but all of them are elongated cylinders having relatively similar shape proportions^8^. Each chromatid of the largest human metaphase chromosome contains ~280 Mb of DNA^9^; this corresponds to a giant DNA molecule of ~9.5 cm that is confined in a cylinder of ~0.6 μm diameter and ~7 μm length^8^.

On the basis of results obtained using different experimental approaches, several models for the folding of the chromatin filaments within mitotic chromosomes have been proposed. From early electron microscopy images of histone-depleted chromosomes, it was suggested that chromatin fibres form radial loops attached to a protein scaffold^10^,^11^. Results obtained *in vivo* with chromosomes containing engineered regions with altered sequence composition^12^, and with chromosomes in different condensation stages^13^, suggested a model based on hierarchical folding of fibres of increasing diameter (from 30 to 250 nm); in this model the chromatids are segmented longitudinally into layers (~250 nm thickness) formed by the thicker fibre. Models considering that the chromatin filament is irregularly folded were proposed from stretching experiments showing that chromosomes do not have a mechanically continuous protein scaffold^14^ and from the analysis of cryo-sections of mitotic chromosomes^15^. More recently, results obtained using chromosome conformation capture methods were interpreted with polymer simulations and it was suggested that compact chromatids are formed by arrays of stochastically positioned chromatin loops that are longitudinally compressed^16^. All these models are based on chromatin fibres that are folded and form a three-dimensional network that fills the chromatid. However, unexpectedly, it was observed that incubation of chromosomes at 37 °C (under metaphase ionic conditions) on electron microscopy grids caused the emanation multilayered plates instead of fibres^17^. Further studies using polarizing microscopy, electron tomography, AFM imaging in aqueous media, and AFM-based nanotribology and force spectroscopy^18^,^19^,^20^ showed that in each layer the chromatin filament forms a flexible two-dimensional network in which nucleosomes are irregularly oriented, allowing the compaction of the structure by interdigitation of adjacent layers (each layer has an apparent thickness of ~6 nm). This led to the proposal of the thin-plate model in which it is considered that chromatids are filled by many stacked layers of chromatin oriented perpendicular to the chromatid axis^18^,^19^. Furthermore, it was demonstrated that chromatin plates can be self-assembled from chromatin fragments obtained by micrococcal nuclease digestion of metaphase chromosomes^21^. The nano- and micro-mechanical data obtained from chromatin and chromosome stretching experiments^22^,^23^,^24^,^25^, and modelling studies^26^,^27^ of other laboratories, were used to estimate the nucleosome-nucleosome interaction energies between the stacked chromatin layers; it was demonstrated that the different energy components of this structure can explain the elongated cylindrical shape and the mechanical properties of metaphase chromosomes^8^.

On the other hand, although cytogenetics is mainly concerned with the study of chromosome structure for clinical diagnostics^28^, there is a wealth of cytogenetic data that may give insights about fundamental aspects of chromosome structure. Mitotic chromosomes show longitudinal heterogeneities that allowed the development of diverse cytogenetic staining procedures that produce banding patters characteristic of each human chromosome^29^,^30^. Each staining method gives rise to a continuous pattern of dark and light bands and sometimes the patterns are complementary: the Giemsa positive bands (dark G-bands) are located in the zones in which the bands obtained with reverse staining methods show a low intensity (light R-bands). Therefore, from a structural point of view bands occupy the whole chromosome. The sequence of the human genome indicated a correlation between regions of low GC content and dark G-bands, and between regions high GC content and light G-bands^9^. The number of bands depends on the compaction degree of the chromosomes; human karyotypes corresponding to mid-prophase chromosomes yield up to 2000 bands^31^, but current analyses are carried out with karyotypes of 400- to 800-band levels^30^. Furthermore, bands can be split into several sub-bands when chromosomes are mechanically stretched^32^. Fluorescence *in situ* hybridization (FISH) allowed the positioning of many genes with respect to the chromosome bands^28^, and it was found that the correspondence between the cytogenetic map and the genome sequence is excellent^9^,^33^. Many structural chromosome rearrangements such as sister chromatid exchange (SCE), and chromosome fusions and translocations have been observed; most of them are related with human diseases^34^. Chromosome painting techniques, in particular multicolour spectral karyotyping (SKY) and multiplex (M)-FISH^35^,^36^,^37^,^38^, permitted the simultaneous discrimination of all human chromosomes and greatly facilitated the study of structural rearrangements. In this work, typical results obtained in different banding experiments and in the analyses of SCE and other rearrangements have been used to obtain information about the internal structure of chromosomes and to test the models considered in the preceding paragraph. It is demonstrated that the model based on stacked layers of chromatin can explain the main geometric characteristics of chromosome bands and translocations.

## Results and Discussion

### Chromosome bands are perpendicular to the chromosome axis

**Figure 1:**
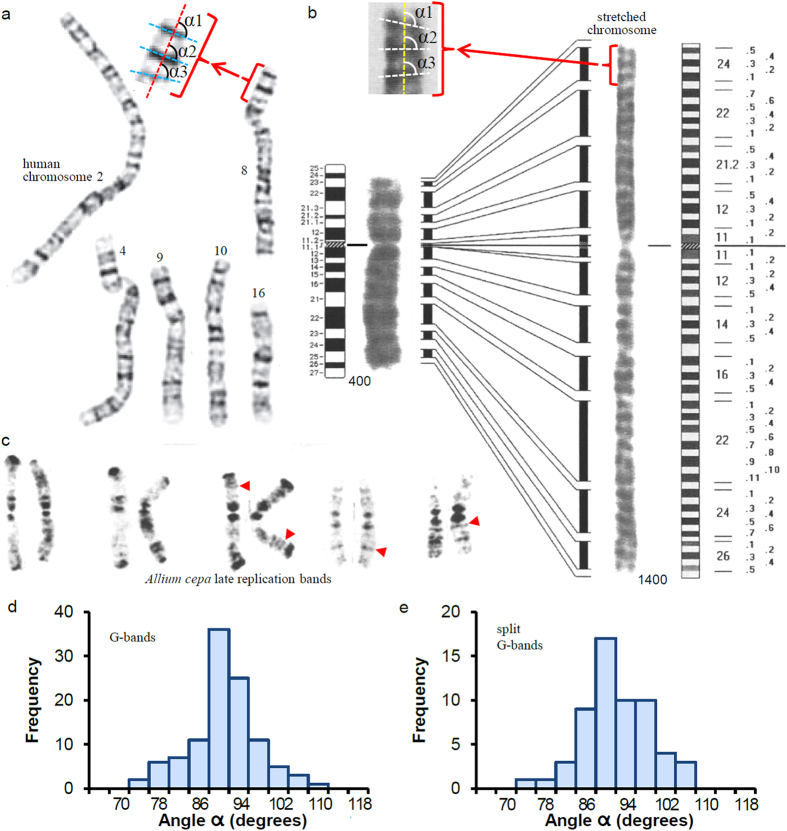
Orientation of chromosome bands with respect to the chromosome axis. The orientation angles (*α*1, *α*2, *α*3…) were measured as schematized in (**a,b)**. (**a)** Example showing chromosome G-banding patterns at ~700-band level. (**b)** Chromosome G-bands and the corresponding ideograms before (~400-band level) and after stretching (~1400-band level). (**c)** Example of replication-banding patterns in which dark-stained bands indicate late-replicating regions; red arrow heads point to thin bands. (**d)** Histogram of the orientation angles of G-bands (~700-band level). (**e)** Orientation angles of G-bands of stretched chromosomes (shown in (**b**)). Figures in (**a**–**c)** have been reproduced, respectively, from refs 39,32,40 with permission.

In the G-banded chromosomes shown in the example presented in Fig. 1a (reproduced from ref. 39), it seems that bands have a transverse orientation. The results of the quantitative measurement (see Methods) of the orientation angle *α* (defined with respect to the chromosome axis) of many bands are presented in Fig. 1d. These results correspond to G-banding patterns at ~700-band level. Equivalent results were obtained with G-banded chromosomes at a lower resolution (~400-band level) and with R-banded chromosomes (see Table 1). The conclusion of this quantitative study is that chromosome bands are orthogonal to the chromosome axis. The ideograms of chromosome banding patters (ref. 30; see Fig. 1b) are schematic drawings that are not based on quantitative measurements of the orientation angle but, in agreement with this conclusion, they represent all the bands exactly perpendicular to the longitudinal axis of the chromosome. Interestingly, even the thinnest bands in the typical karyotypes (Fig. 1a) and in the corresponding ideograms^30^ show this orientation and occupy the entire cross-section of the two sister chromatids. Since the analysis of the human genome showed that the thinnest bands correspond to sequences of less than 1 Mb^9^, this result indicates that within metaphase chromosomes chromatin containing relatively short stretches of DNA fills completely the cross-section of each chromatid. Furthermore, as top and bottom boundaries of thin bands are usually well defined, in three dimensions bands can be considered disc-like structures. All these results suggest that the chromatin filament must be able to fold forming very short cylinders having the same diameter as the chromatids and the same height as the thin bands. It will be shown below that many proposed models for chromosome structure do not have the adequate properties to satisfy these geometrical requirements.

### Maintenance of the transverse orientation of bands after their mechanical splitting

The images from ref. 32 reproduced in Fig. 1b show that chromosome stretching causes band splitting. It can be seen that many of the resulting split bands are well defined and relatively thin. The measurements of the orientation angle *α* of these bands are presented in Fig. 1e. The mean of these measurements is about 90° (Table 1), indicating that splitting does not change the orthogonal orientation of bands with respect to the chromosome axis. In three dimensions such a transformation leading to defined sub-bands can only be explained considering that chromatin structure within bands is formed by planar elements that can be relatively easily pulled apart by stretching forces. It will be discussed below that many structural models of metaphase chromosomes are not compatible with these observations.

### Perpendicular orientation of thin replication bands with respect to the chromosome axis

Replication bands were observed in many different types of eukaryotes^29^. The example shown in Fig. 1c (reproduced from ref. 40) corresponds to replication bands of plant chromosomes. In this experiment rood cells were treated with 5-bromodeoxyuridine and the resulting chromosome spreads were stained with Giemsa; dark bands are produced by late-replicating regions. The measurements of the orientation angle *α* of replication bands of two different species (see Table 1) indicate that they are orthogonal to the chromosome axis. The well-defined thin bands observed in these preparations (some of them are indicated with red head arrows in Fig. 1c) are particularly interesting because each one contains a short segment of DNA corresponding to different late-replicating sequences. This observation indicates again that chromatin containing a relatively short stretch of DNA can form a disk-like structure that occupies the whole cross-section of the chromatid. These results reinforce the conclusions obtained above from the analysis of thin bands in typical human karyotypes.

### In the reciprocal exchanges between sister chromatids the resulting connection surfaces are flat and perpendicular to the chromatid axis

**Figure 2:**
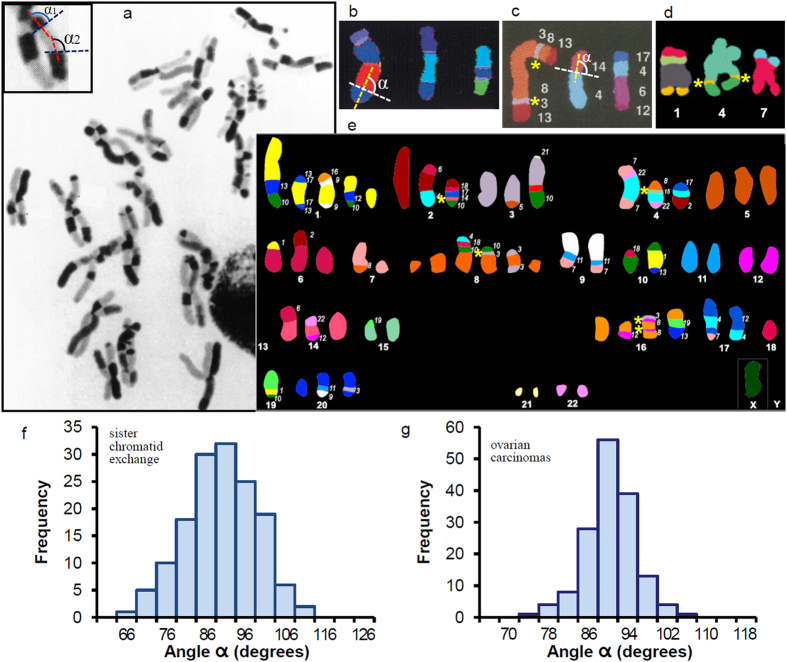
Orientation of connection surfaces in translocations and other chromosome rearrangements. The orientation angles (*α*, *α*1, *α*2…) were measured as schematized in (**a**–**c)**. (**a)** Examples of SCE in *A. cepa*. Examples of dicentric chromosomes in malignant fibrous histiocytoma (**b**) and of translocations in HeLa cells (**d**) visualized by M-FISH. Examples of translocations identified by SKY corresponding to breast (**c**) and ovarian (**e**) carcinomas. The yellow asterisks in (**c**–**e**) indicate thin translocated chromosome segments. (**f)** Histogram of the orientation of the connection surfaces observed in SCE. (**g)** Orientation angles of the connection surfaces found in ovarian carcinomas. Figures in (**a**,**c**,**d)** have been reproduced, respectively, from refs 41,35,42 with permission. Figure (**b)** has been reproduced from ref. 61, Copyright (2000) National Academy of Sciences, USA. Figure (**e)** (OVCAR-8 cell line) has been reproduced from http://home.ncifcrf.gov/CCR/60SKY/new/demo1.asp (Cell Line NCI60 Drug Discovery Panel^58^).

The images of SCEs shown in the example presented in Fig. 2a (reproduced from ref. 41) were obtained using different proportions of 5-bromodeoxyuridine to deoxythymidine in three successive cell division cycles, followed with Giemsa staining of the resulting metaphase spreads. Using this procedure chromatids acquire different staining degrees that allow the visualization of the interchanged chromatid segments. Note that the boundaries between the interchanged segments and the original chromatids are sharp and well defined, indicating that the connection of the two chromatids is produced in a very narrow zone. The results obtained in the measurements of the orientation angle *α* (with respect to the chromatid axis) of these boundaries are shown in Fig. 2f; the mean value of angle *α* is 90° (Table 1). Taken together, these results indicate that in three dimensions the connection of the interchanged and original chromatids is produced in a thin planar zone oriented perpendicular to the chromatid axis. To be acceptable, a structural model of the metaphase chromosome must be consistent with these observations (see below).

### The connection surfaces of the multiple translocations occurring in cancer cells are planar and perpendicular to the chromosome axis

Several examples of chromosome rearrangements in different malignancies are shown in Fig. 2b–e. These images were obtained using SKY and M-FISH techniques in different laboratories (see references in the figure legend); each chromosome was painted with a different colour to clearly identify fusions leading to dicentric chromosomes (b) and translocations (c-e). It seems that both fusion and translocation junctions have a transverse orientation. The same orientation is observed in translocations associated to different non-oncological diseases^35^, but the many rearrangements observed in cancer cell karyotypes facilitate the study of the morphology of translocated chromatids. The results obtained in the quantitative measurement of the orientation angle *α* with respect to the chromosome axis of the translocation junctions of four human ovarian carcinoma cell lines (the SKY karyotype of one of them (OVCAR-8) is shown in Fig. 2e) are presented in Fig. 2g. The quantitative analysis of many translocations corresponding to different cancer cells (pancreatic, skin, and cervical carcinomas, and leukaemia and other haematological malignancies) led to equivalent results (see Table 1). In most of the images obtained using chromosome painting methods, the sister chromatids of each metaphase chromosome are closely joined together (Fig. 2), but in some cases the two sister chromatids are visible. In particular, M-FISH karyotypes of cervical carcinoma HeLa cells (ref. 42; reproduced in part in Fig. 2d) allowed the measurement of the orientation angle of the translocation connection surfaces of chromosomes with sister chromatids longitudinally joined and with single chromatids visible. The results obtained in both cases are equivalent (Table 1). The conclusion from all these measurements is that the angle *α* of translocation junctions is approximately 90°. Furthermore, many junctions are sharp and well defined (see examples in Fig. 2b–e), indicating that in three dimensions the connection between the original chromatid and the translocated part is a thin planar region orthogonal to the chromatid axis. Even when the translocations are very small (see examples in Fig. 2c–e indicated with yellow asterisks), the top and bottom boundaries between the translocated segment and the original chromatid are usually very well defined. This indicates that the relatively short sequences of genomic DNA corresponding to the small translocated chromatid segments are confined within thin discoidal structures. All these observations reinforce the conclusions obtained in the preceding sections and impose geometric constraints that have to be considered for the validation of models for chromatin folding in metaphase chromosomes.

### The geometric characteristics of chromosome bands and rearrangements provide a test for the different models proposed for chromatin folding within mitotic chromosomes

**Figure 3:**
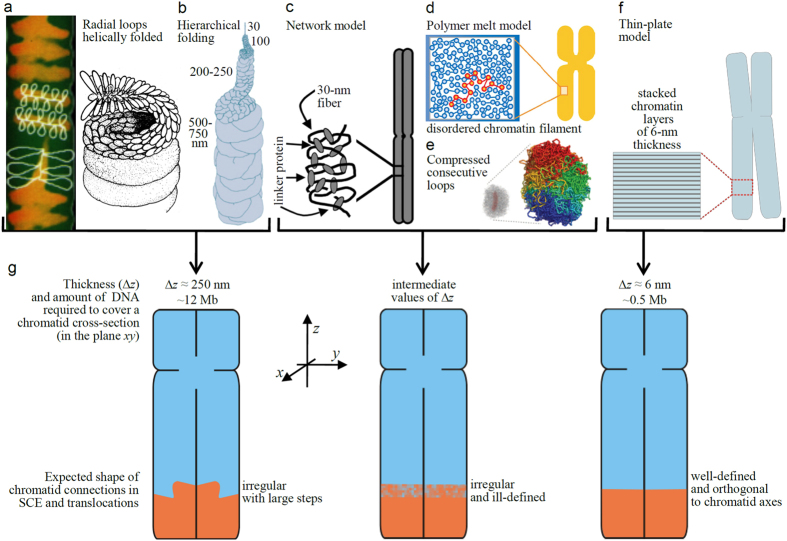
Representation of the expected shape of chromatid connections in SCEs and translocations for different chromosome structural models. The amount of DNA required to cover completely a chromatid cross-section is different for each chromosome model (**a–f**) and this determines the shape of the chromatid connections in rearrangements (**g**). For the three categories schematized in (**g**), the amount of DNA necessary to cover the chromatid cross-section and the corresponding thickness of the connection zone (Δ*z*) were calculated for human chromatids having a DNA density of 166 Mb/μm^3^ and a diameter of 0.6 μm^8^,^62^. The models schematized in figures (**a**, left), (**a**, right), (**e**), and (**f**) have been reproduced, respectively, from refs 44,11,16,8 with permission. Model in figure (**b**) has been obtained from Fig. 7B(**a**) of ref. 13, ©2004 Kireeva *et al*. The Journal of Cell Biology, 166: 775-785, doi:10.1083/jcb.200406049 (http://jcb.rupress.org/content/166/6/775.short). Figures (**c,d**) have been reproduced, respectively, from refs 14,15, Copyright (2002) and (2008) National Academy of Sciences, USA.

The measurements of the orientation angle *α* of cytogenetic bands, replication bands, and of the connection surfaces in SCEs and in translocations indicate that in all cases they are orthogonal to the chromatid axis. It will be shown below that this is consistent with the basic geometric features of the thin-plate model (Fig. 3f; refs 8,18, 19, 20, 21). However, these measurements show a variability (standard deviation 5.0–8.9^o^; Table 1) that at least in part could be attributed to the soft structure of chromosomes; chromosomes are hydrogels with good mechanical properties but they are easily deformable^8^,^25^. This reduces the capability of these measurements for direct discrimination between different chromosome models, but the perpendicular orientation of bands and connection surfaces, combined with other cytogenetic results, can be used to test different proposals for metaphase chromatin folding.

As indicated above, genomic results have shown that the thinnest bands contain less than 1 Mb of DNA^9^ and that there is an excellent correspondence between band location on a given mitotic chromosome and its position on the DNA sequence corresponding to this chromosome^33^.Therefore, the DNA molecule within each chromatid is organized in such a way that it can fill consecutively (without overlapping) the successive bands from one telomere to the other. According to the results presented in the preceding sections, the well-defined discoidal shape and orthogonal orientation of the thinnest bands indicate that DNA stretches of less than 1 Mb can fill short cylinders having the diameter of the chromatid. In agreement with this, FISH results showing that sequences separated by 0.5–1 Mb frequently produce hybridization spots on either side of the chromatid suggested many years ago that short stretches of DNA can cross the width of the chromatid^43^. The thin discoidal shape and orthogonal orientation of thin replication bands (Fig. 1c) and of small translocated chromatid segments (Fig. 2c–e) are also in agreement with these observations. Chromosome structural models consistent of radial loops helically folded (Fig. 3a; refs 10,11,44) and the hierarchical folding model (Fig. 3b; refs 12,13) are not compatible with these results because they need a large amount of DNA (~12 Mb) to span the cross-section of a chromatid (Fig. 3g, left scheme); this corresponds to a chromatid region having a thickness (Δ*z*) of ~250 nm. The amount of DNA required to cover the cross-section is reduced to ~3 Mb if it is hypothesized^45^ that the radial loops of the 30-nm chromatin fibre are organized forming minibands. In the thin-plate model (Fig. 3f; refs 8,18, 19, 20, 21), only a monolayer of nucleosomes is needed to cover the cross-section of a chromatid. This corresponds to ~0.5 Mb of DNA (Fig. 3g, right scheme); furthermore, layers are interdigitated and have a small thickness^18^,^19^ (Δ*z* ≈ 6 nm). In the network (Fig. 3c; ref. 14), polymer-melt (Fig. 3d; ref. 15), and compressed loop (Fig. 3d; ref. 16) models the path of the chromatin filament is irregular and the length of DNA required to cover completely a chromatid cross-section and Δ*z* are larger than the values corresponding to the thin-plate model; the models in Fig. 3c–e cannot justify the existence of thin bands containing less than 1 Mb of DNA^9^.

The thin-plate model is consistent with the experimental observations considered in the preceding paragraph and it can justify the results obtained in FISH experiments with metaphase chromosomes showing that the resolution of probe ordering is 1–3 Mb^46^. In contrast, in the models schematized in Fig. 3a–e, the path of the chromatin filament with respect to the z axis (Fig. 3g, left and central scheme) constantly goes up and down, making difficult the correlation between the order of probes in the metaphase chromosome and the DNA sequence. For the same reasons, with these models it is difficult to explain the band splitting observed in chromosome stretching experiments, but in the thin-plate model the relative weakness of the interactions between the stacked chromatin layers^8^,^18^,^19^ can justify easily the observed band splitting and the maintenance of the orthogonal orientation of the resulting split bands (Fig. 1b,e and Table 1).

In SCEs and in translocations the connection between the original chromatid and the exchanged segment is planar and perpendicular to the chromatid axis. This geometry is not at all compatible with the very uneven shape of chromatid connections (Fig. 3g, left scheme) expected for the models of radial loops and of hierarchical folding (Fig. 3a,b; refs 10, 11, 12, 13,44). In the network (Fig. 3c; ref. 14) and polymer-melt (Fig. 3d; ref. 15) models the chromatin filament is irregularly folded and consequently well-defined flat connections are not expected (Fig. 3g, central scheme). Chromatin folding in these models is isotropic, making difficult the explanation of the orthogonal orientation of the junction surfaces with respect to the chromatid axis. The model consisting on a longitudinally compressed array of consecutive chromatin loops^16^ has a certain degree of longitudinal order but, as can be seen in Fig. 3e, the irregular folding of the chromatin fibre considered in this model cannot justify the well-defined planar connection surfaces of translocations and other rearrangements. In contrast, the thin-plate model is anisotropic^8^,^18^,^19^; the stacked layers perpendicular to the chromatid axis (Fig. 3f) can explain the planar geometry and orthogonal orientation (Fig. 3g, right scheme) of the connection surfaces observed in SCEs and translocations.

**Figure 4:**
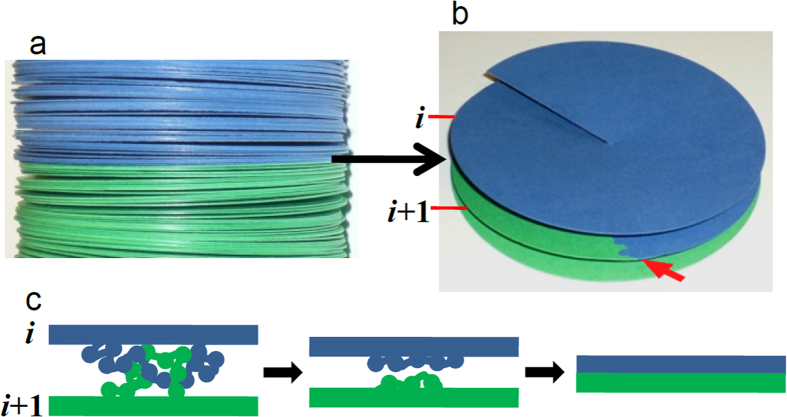
Simplified solid model representing part of a chromatid with many stacked layers. (**a**) Representation of a translocation as seen in SKY and M-FISH experiments: the original chromatid (blue) is joined to a translocated chromatid (green). (**b**) This less compact representation illustrates that the connection with the translocated chromatid occurs in a defined region (pointed by a red arrow). (**c**) If some entanglement of DNA is produced between two consecutive turns (*i* and *i* + 1), topoisomerase II could regenerate the disentangled uniform structure.

The simplified model in Fig. 4a illustrates the three-dimensional compatibility of the multilayer organization of chromatin with the geometric characteristics of translocations. It can be seen that the connection surface between the original chromatid and the translocated part is well defined and perpendicular to the chromatid axis. The well-defined top and bottom surfaces often observed in chromosome bands can also be explained with this model. Different experimental results indicated that the metaphase chromatids are helical structures^11^,^47^,^48^,^49^ and it was suggested that chromatin layers are connected forming a continuous helicoid^8^,^19^ with the successive turns in close contact (helicoidal pitch of ~6 nm). The detailed path of DNA in this continuous structure is unknown but, as schematized in Fig. 4b, the junction between the two chromatid fragments should be produced abruptly at the point in which the DNA of the translocated part is covalently bound to the DNA of the original chromatid. Therefore, since the thickness of chromatin sheet in the helicoidal turns is very small, this model justifies that the transition between the translocated and original chromatid is produced in a very narrow zone of the *z* axis, in agreement with the experimental observations. This continuous helicoidal folding permits a simple topological organization of DNA without entanglements. The absence of topological entanglement between adjacent layers is fully compatible with the band splitting observed in chromosome stretching experiments. For the maintenance of this organization, it has to be taken into account that during mitosis there is a high topoisomerase II activity^50^, which allows DNA segments in the chromatin filament to pass through each other^51^. Although this activity is mainly related with the disentanglement of DNA between sister chromatids^52^, if entanglements are produced within a chromatid (see drawing in Fig. 4c), it can be speculated that topoisomerase II could also be involved in the disentanglement of chromatin turns in condensed chromosomes. It seems difficult to explain any enzymatic activity inside compact chromatids. However, metaphase chromosomes are fluid and dynamic structures that can be considered as hydrogels with a lamellar liquid crystal organization^8^; they are highly hydrated^53^ and it has been found^54^,^55^ that in living cells topoisomerase II can rapidly diffuse within metaphase chromatin.

### Biomedical implications

In addition to give an explanation to all the geometric characteristics of chromosome bands and rearrangements, it was shown previously^8^ that the thin-plate model can explain the morphology and dimensions of metaphase chromatids. The energy components of this multilayer organization can also justify the outstanding mechanical properties of metaphase chromosomes^8^,^20^,^25^.

The multilayer organization of chromatin in metaphase chromosomes was proposed from experimental studies in which native chromosomes and plates were prepared in aqueous solutions containing metaphase cation concentrations^17^,^18^,^19^,^20^,^21^. In particular, in cryo-electron microscopy and AFM experiments^18^,^20^, water solutions where present even during the imaging of plates. In contrast, in the typical metaphase cell spreads used in cytogenetic studies chromosomes are fixed with methanol-acetic acid^38^,^39^,^46^. Early results showed that these organic solvents remove part of the histones^56^. However this does not imply that the multilayered organization of native chromosomes disappears when chromosomes are fixed with methanol-acetic acid. In fact, the observation of orthogonal and well-defined thin bands and orthogonal and smooth surface connections in SCEs and translocations indicates that the backbone of this multilayered structure is maintained after fixation. Thus, although methanol-acetic acid fixation may extract histones, this treatment preserves the global structure of the chromosome and its multilayered organization. This could explain the well-defined images of chromosome bands and structural rearrangements obtained in many laboratories using different cytogenetic procedures.

This study has tried to build a bridge between results obtained with chromosomes in aqueous media and results obtained with chromosomes fixed with menthol-acetic acid. It has been shown that a coherent synthesis is possible. The conclusions of this research can be useful for a better understanding of the structure of native chromosomes and of the structural basis of the cytogenetic methods considered in this work. The impact of clinical cytogenetic analyses on the diagnosis of hereditary diseases and cancers is impressive^28^,^57^. This work provides for the first time a consistent explanation of the chromosome structural elements on which these analyses are based.

## Methods

The measurements of the orientation angle *α* of well-defined thin G-bands with respect to the chromosome axis were obtained from human karyotypes at ~400-band level (Fig. 6 of Shaffer *et al*.^30^) and ~700-band level (Fig. 2 of Venter and coworkers^39^; reproduced in part in Fig. 1a). Angle *α* is defined as indicated in Fig. 1a: the chromosome axis is equivalent to the median line (dotted red line); the dotted blue line was drawn according to the observed orientation of each analyzed band. The same procedure was used for the measurement of angle *α* of R-bands of human chromosomes (Fig. 3 from ref. 30). Images obtained after stretching of human chromosome 6 (Figs 1 and 3 of Hliscs *et al*.^32^; partially reproduced in Fig. 1b) were used to measure the orientation of split G-bands; angle *α* of these bands in homogeneously stretched regions was measured as indicated in Fig. 1b. Replication banding patters of *Allium cepa* and *A. nigrum* (Figs 1 and 4 of Cortés and Escalza^40^; partially reproduced in Fig. 1c) were used to measure the orientation angle *α* of thin bands corresponding to late-replicating regions. In the rearrangements produced by exchanges between sister chromatids, the orientation angle *α* of the connection surfaces with respect to the chromatid axis was measured (as indicated in Fig. 2a) from images obtained in SCE experiments with *A. cepa* (Figs 9.3 and 9.5 of Friebe and Cortés^41^; reproduced in part in Fig. 2a). Angle *α* of the connection surfaces between a translocated chromatid and the original chromatid was measured as shown in Fig. 2b,c. These measurements were obtained from SKY images of (i) ovarian carcinomas (OVCAR-3, 4, 5, and 8 cell lines^58^; OVCAR-8 is reproduced in Fig. 2e), (ii) pancreatic carcinomas (AsPc-1, Capan-1 and 2, Colo-357, FA6, MDA Panc-3, PaTu II, SUIT-2, and T3M-4 cell lines^59^), and (iii) haematological malignancies (Figs 1, 2, 3 of Ried and coworkers^37^). Angle *α* of translocations seen in M-FISH karyotypes was obtained from images corresponding to (i) skin carcinomas (Fig. 2 of Popp *et al*.^60^) and (ii) HeLa cells (Fig. 3 of Landry *et al*.^42^; partially reproduced in Fig. 2d). A fraction (~7%) of the analyzed translocations appeared very distorted and were not used in these measurements. In all cases angle *α* was measured with the angular dimension tool of CorelDRAW X3 software.

## Additional Information

**How to cite this article**: Daban, J.-R. Stacked thin layers of metaphase chromatin explain the geometry of chromosome rearrangements and banding. *Sci. Rep*. **5**, 14891; doi: 10.1038/srep14891 (2015).

## Figures and Tables

**Table 1:** Orientation of chromosome bands and of connection surfaces in translocations and other rearrangements.

Banding and rearrangements^a^	Orientation angle (*α*)^b^ with respect to thechromosome axis
G-banding in human chromosomes ~500-band level ~700-band level	90.1 ± 5.2 (*n *= 38)91.1 ± 6.7 (*n *= 107)
R-bands in human chromosomes	88.4 ± 8.1 (*n *= 23)
Split G-bands (stretched chromosomes)	92.0 ± 6.7 (*n *= 58)
Replication bands *A. cepa* *A. nigrum*	90.6 ± 6.0 (*n *= 18)90.1 ± 6.2 (*n *= 15)
SCE in plant chromosomes	90.0 ± 8.9 (*n *= 148)
Translocations seen by SKY Ovarian carcinomas Pancreatic carcinomas Haematological malignancies	90.6 ± 5.0 (*n *= 154)89.9 ± 8.3 (*n *= 142)90.9 ± 5.4 (*n *= 30)
Translocations in M-FISH karyotypes Skin carcinomas HeLa cells Sister chromatids closely joined Two sister chromatids visible	89.5 ± 5.0 (*n *= 25)90.3 ± 5.6 (*n *= 26)91.6 ± 8.8 (*n *= 20)

^a^G- and R-banded karyotypes, stretched chromosomes, replication-banding patterns, SCE images, and M-FISH and SKY-painted chromosomes used for the measurements presented in this table are indicated in Methods.

^b^Angle *α* (in degrees) corresponding to chromosome bands, SCEs, and translocations is defined in Figs 1 and 2. Values shown are means ± SD of the indicated number (*n*) of independent measurements.
